# QTL analysis of cocoon shell weight identifies BmRPL18 associated with silk protein synthesis in silkworm by pooling sequencing

**DOI:** 10.1038/s41598-017-18277-y

**Published:** 2017-12-21

**Authors:** Chunlin Li, Xiaoling Tong, Weidong Zuo, Yue Luan, Rui Gao, Minjin Han, Gao Xiong, Tingting Gai, Hai Hu, Fangyin Dai, Cheng Lu

**Affiliations:** 1 State Key Laboratory of Silkworm Genome Biology, Southwest University, Chongqing, 400716 China; 2 Key Laboratory of Sericulture biology and Genetic breeding, Agricultural Ministry, College of Biotechnology, Southwest University, Chongqing, 400716 China

## Abstract

Mechanisms that regulate silk protein synthesis provide the basis for silkworm variety breeding and silk gland bioreactor optimization. Here, using the pooling sequencing-based methodology, we deciphered the genetic basis for the varied silk production in different silkworm strains. We identified 8 SNPs, with 6 on chromosome 11 and 1 each on chromosomes 22 and 23, that were linked with silk production. After conducting an association analysis between gene expression pattern, silk gland development and cocoon shell weight (CSW), *BMGN011620* was found to be regulating silk production. *BMGN011620* encodes the 60S ribosomal protein, L18, which is an indispensable component of the 60S ribosomal subunit; therefore we named it *BmRPL18*. Moreover, the clustering of linked SNPs on chromosome 11 and the analysis of differentially expressed genes reported in previous Omics studies indicated that the genes regulating silk protein synthesis may exhibit a clustering distribution in the silkworm genome. These results collectively advance our understanding of the regulation of silk production, including the role of ribosomal proteins and the clustered distribution of genes involved in silk protein synthesis.

## Introduction

The ability of silkworm to synthesize silk proteins has laid the foundation for the sericulture industry that has contributed greatly to the economy of different countries such as China, Brazil and India^1^. Current molecular technologies have not only advanced the understanding of silk protein regulation in silkworm but have also promoted the use of these lepidopterans as efficient bioreactors. A number of studies have reported the possibility of using silkworm as a bioreactor to produce exogenous proteins^2–6^. Therefore, understanding the regulatory mechanism and identifying genes controlling silk protein synthesis will be of great significance to breed silkworm strains that yield high quantities of silk and to optimize bioreactor parameters.

Cocoon related traits, such as cocoon shell weight (CSW) and cocoon shell ratio (CSR) are the main phenotypes that reveal the ability of a silkworm strain to synthesize the main silk proteins, fibroins and sericins. These traits were shown to be quantitative and to have complex genetic basis indicating an intricate regulatory mechanism underlying silk protein production^7–10^. Despite several mapping analysis conducted to decipher the genetic architecture of silk production related traits^8,11–13^, the identification of genes regulating silk production remains elusive because of the unavailability of marker maps with high density, complex genetic basis, special genetic characteristics, etc.

In this study, we combined next generation sequencing and bulked segregation analysis (BSA) to identify the genes that control silk protein synthesis. This methodology, termed as pooling-sequencing, is a newly developed protocol to identify genes with high efficiency by combining individuals with similar phenotypes for high coverage sequencing of genes or regions associated with traits of interest^14,15^. Application of pooling sequencing has facilitated the rapid development of high density SNP maps at a relatively low cost^16^. Currently, several derived methods based on pooling sequencing, such as Mutmap sets^17,18^ and QTL-Seq etc^19^, have been widely used to identify candidate genes that regulate qualitative traits, quantitative traits and domestication mechanisms in crops and livestock^20^. However, these methodologies have shown some limitations, such as sequencing errors and allele ratio bias that attribute to differential representation of individuals in the pools and deficient sequencing depths, which may cloud screening of candidate genes based on the associated markers. Thus, experiments, including allele ratio confirmation by individual genotyping, gene expression pattern analysis, and functional validation are necessary for the identification of candidate genes.

In this study, we report pooling sequencing for the first time to screen the associated SNPs that regulate silk protein synthesis in *B. mori*. Then, using allele ratio confirmation by individual genotyping, EST counts analysis of adjacent genes and expression pattern analysis of candidate genes, we identified *BMGN011620* that may be one of the genes controlling silk protein synthesis. Since *BMGN011620* encodes the 60S ribosomal protein, L18, in silkworm we term it *BmRPL18*. Based on a comprehensive analysis of this study and previous Omics data, we propose for the first time that genes regulating silk protein synthesis may be distributed in clusters in the silkworm genome.

## Results

### The pooling sequencing-based methodology

In order to identify the genes regulating silk protein synthesis, we integrated several gene screening methods to incrementally identify target genes. These methods included a BSA-based linkage mapping, putative associated SNP confirmation, EST data analysis of genes in the mapping regions and association analysis of gene expression patterns. The protocol used in this study is illustrated in the Supplementary Figure 1. First, the silkworm strains with huge variations in silk synthesis were selected as parents to produce the F_1_ generation, and the F_1_ males were backcrossed with the parent strain yielding lower silk to produce the mapping population (BC_1_M). Then, pools were made by segregating individual yielding extremely low or extremely high silk production from each moth area. Thereafter, the pools and the parents were used to develop a high density SNP map and to conduct the linkage analysis through standard specific length amplified fragment (SLAF) sequencing. Because of the occurrence of false positives in the BSA methodology, the linked relationship of the putative linked SNPs was confirmed by individual genotyping. Based on the resulting positive linked SNPs, we defined the linked region to 300 kb regions around these positives (the approximate physical distance per cM in silkworm). Then, genes in these regions were screened based on their EST data. Since silk is synthesized in the silk glands, genes with EST representation in silk gland were selected for the association analysis of expression pattern to identify the genes associated with silk synthesis regulation. ESTs expressed in the embryo were also obtained for the analysis.

### Overview of sequencing pools and SLAF-tags development

**Figure 1:**
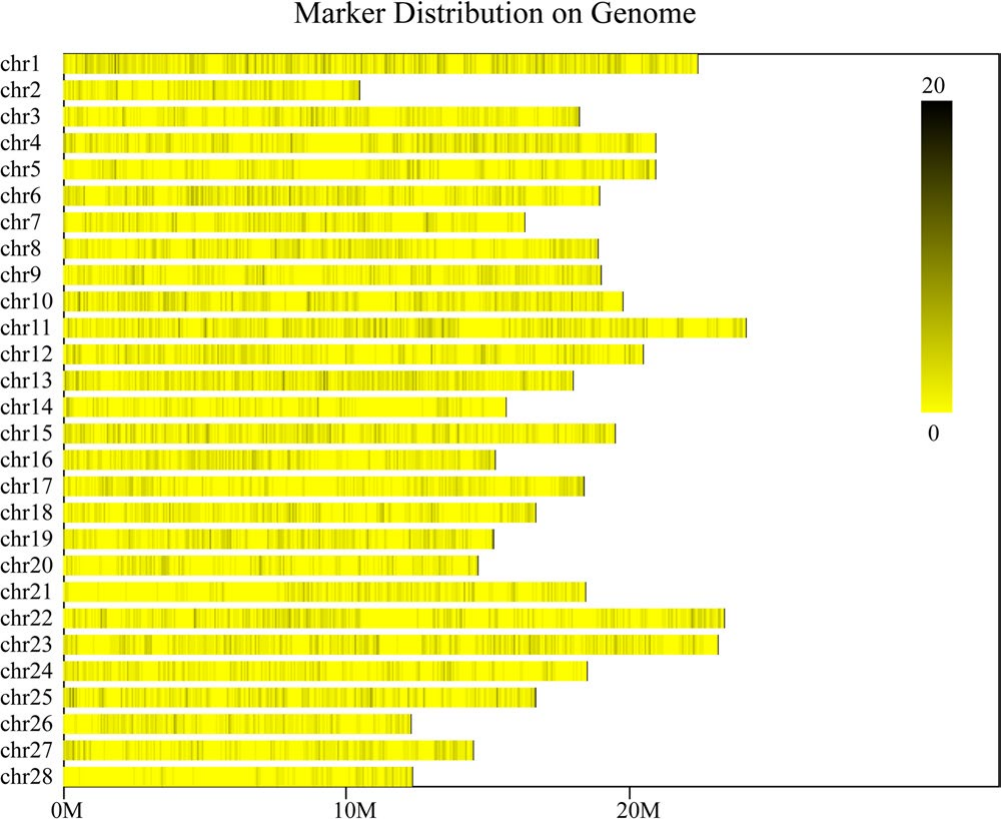
Distribution of polymorphic SLAF-tags on each chromosome in Bombyx mori. The lateral bars represent the chromosomes of silkworm and black vertical lines on them means the SLAF-tags.

Two silkworm strains, inbred Dazao (IS-Dazao) and 872B, with huge variations in silk yield, represented by the CSW, were selected as parents to produce the mapping population (BC_1_M). The population comprised 7 moth areas and each contained 127 to 174 male individuals (Supplementary Table 1). Note that only male individuals were used to avoid the interference of gender effect^7^. We surveyed the male offspring’s CSW from each pair of parents and found only a slight difference in the average performance (Supplementary Figure 2) among the 7 pairs, which may have resulted from the different population densities. Thus, to accurately select individuals with extreme phenotypes from the BC_1_M population, the offspring derived from each parent pair were selected and mixed into two pools (the L-pool and H-pool in Supplementary Figure 1). Both pools and their parents were then subjected to standard SLAF sequencing. The total sequencing yield was 57.10 M reads (11.14Gbp), which were developed into 122,217 SLAF-tags, with an average depth of 245×. These SLAF-tags were aligned with the silkworm reference genome and mapped to the 28 silkworm chromosomes. We found that the number of SLAF-tags on each chromosome varied from 2,285 to 6,081 (Supplementary Table 2). Notably, SLAF-tags map showed an uneven distribution on the genome (Supplementary Figure 3). Analysis of regions lacking the SLAF-tags revealed that they were rich in repeat sequences, which is presumed to lack information and therefore was artificially filtered by the proper selection of endonuclease combination. Among these SLAF-tags, 12,476 were identified to be polymorphic. From these, 9,143 polymorphic SLAF-tags (Supplementary Table 2) with coverage more than 5× were selected to construct a high quality SLAF-tag map (Fig. 1) for the linkage analysis.

### Linkage analysis and linked region definition

**Figure 2:**
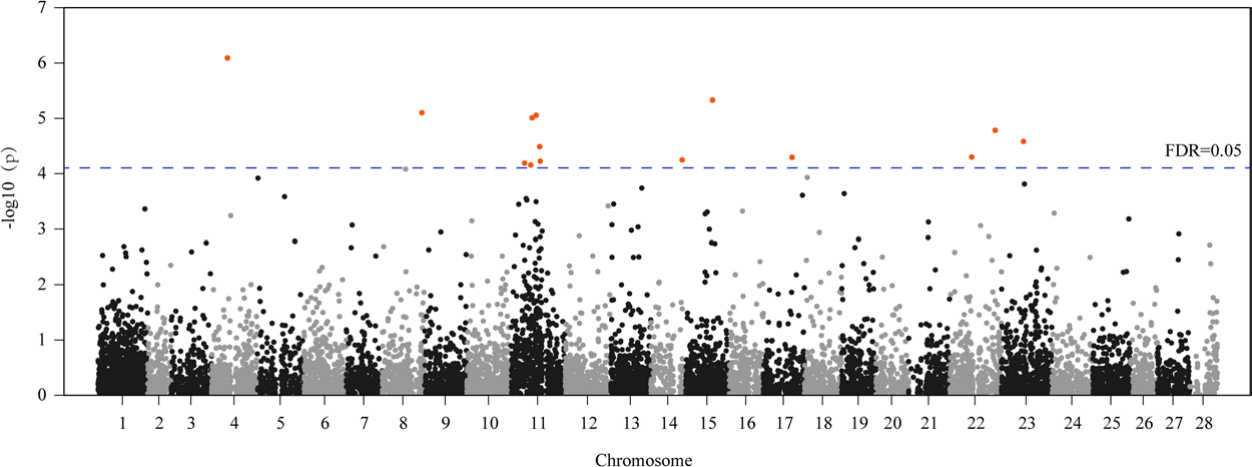
Genome wide linkage analysis based on SNP_index. Blue line shows the significance threshold (FDR = 0.05); the linked markers are highlighted in red.

To identify the SLAF-tags linked with CSW or silk protein synthesis, the linkage analysis was conducted. First, SNP-index for each polymorphic SLAF-tag was calculated based on their genotype frequency in the pools. Then, a Z-test was performed to transform the SNP-index into linked significance (p value). Based on the significance, the false discovery ratio (FDR) of each SLAF-tag was calculated by Bonferroni correction. The SLAF-tags with FDR < 0.05 were selected as the linked markers. In this manner, 14 SLAF-tags were identified to be linked with silk protein synthesis (Fig. 2). One of the system limitations of this methodology is the bias of allele frequency in the sequenced pools. Therefore, it is necessary to confirm the linkage significance of the identified linked SLAF-tags or putative linked SLAF-tags, to avoid false positives caused by the bias. For this, we searched the Indels within 15 kb around these SLAF-tags and determined their genotype in the population for pooling individuals. This confirmed the positive linkage of 8 SLAF-tags with silk synthesis while the remaining 6 were false discoveries (Table 1). Note that among the linked markers, 6 located on the chromosome 11 and 1 each on chromosomes 22 and 23 (Supplementary Figure 4A). Because one only linked marker on the two regions make it weak to define them QTLs, we searched the Indels around the two markers and surveyed whether the flanking markers linked with loci for silk production. And the result showed that the two regions were indeed the QTLs which occupy a series linked markers (Supplementary Figure 4A). Based on the positive linkage of SLAF-tags, we defined the linkage regions to the 300 kb around these positives (the approximate physical distance per cM in silkworm) which is the basis for the following gene screening.

**Table 1:** Confirmation of the putative linked SLAF-markers by the linked indels.

SLAF-marker^1^	Marker(Indel)^2^	Z_value(BSA)^3^	Z_value(Indel)^4^	p_value(BSA)^5^	p_value(Indel)^6^
Marker1453307	M1-1	4.931455366	2.203548955	8.16192E-07	0.02755608
Marker442659	M2-3	−4.465945963	−0.281508006	4.71822E-06	1.221679211
Marker647635*	M3-2	4.419819573	10.63175727	7.97157E-06	0
Marker634361*	M4-1	4.442610549	11.12072674	8.88739E-06	0
Marker1331376	M5-8	4.576942226	3.486750203	9.87833E-06	0.000488928
Marker374140*	M6-8	4.305839786	4.839172679	1.66353E-05	1.30381E-06
Marker193648*	M7-4	4.202642307	5.768207629	2.63817E-05	8.01191E-09
Marker683605*	M8-1	4.153511669	10.91958865	3.27412E-05	0
Marker644146*	M9-1	4.012944613	11.68560395	5.05806E-05	0
Marker299828	M10-5	4.025286803	−1.337554725	5.11301E-05	1.818958368
Marker802294	M11-4	4.050400274	1.130195268	5.69059E-05	0.258393953
Marker339700	M12-6	4.052928074	2.937266818	5.9966E-05	0.003311191
Marker703812*	M13-1	3.99351624	11.05130924	6.51006E-05	0
Marker628661*	M14-1	3.975424118	10.77284357	7.02539E-05	0

*The markers with “*” are the positive linked SLAF-markers.

^1^The putative linked SLAF-markers;

^2^The Indel closely linked with the corresponding putative linked SLAF-markers for linkage confirmation;

^3^The Z_ value of the putative linked SLAF-markers from BSA SLAF sequencing;

^4^The Z_ value of the Indel confirmation;

^5^The p_ value of the putative linked SLAF-markers from BSA SLAF sequencing;

^6^The p_ value of the Indel confirmation.

### Screening for associated genes based on expression pattern

**Figure 3:**
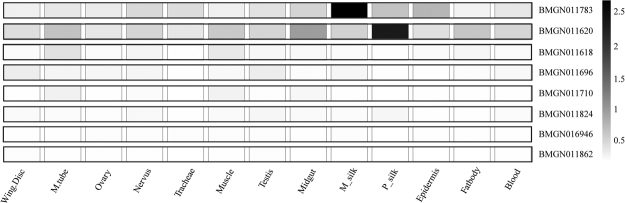
Confirmation of the spatial expression pattern of selected genes. M_tube is short for Malpighian tubes; M_silk and P_silk means the middle silk gland and the posterior silk gland respectively.

In total, 106 genes were predicted in these linkage regions. In order to identify candidate genes, we determined the EST counts of each gene by searching the Kaikobase (http://sgp.dna.affrc.go.jp/KAIKObase/). During silkworm development, two stages critically determine the amount of silk protein synthesized; the embryo and the 5^th^ instar larval stage. Consequently, 11 genes with ESTs present in both 5^th^ instar larval silk gland and embryo were selected as candidates (Supplementary Figure 5). We then surveyed the molecular functions of these genes annotated in databases such as Kaikobase, silkDB and NCBI. Among them, 7 were annotated as genes with known as well important functions, including essential components of respiratory chain, histone modification, autophagy related protein and component of ribosome (RPL18) (Supplementary Table 3). Besides, *BMGN011710*, a gene annotated as TRM112-like protein with no assigned biological functions, had high EST counts in both the 5^th^ instar larval silk glands and the embryo. These 7 genes with known functions as well as *BMGN011710* were chosen for subsequent quantitative RT-PCR and gene expression pattern analyses. The spatial expression pattern analysis in the various silkworm tissues revealed that *BMGN011620* and *BMGN011783* had ubiquitous expression in various tissues with high expression in post and anterior-middle silk glands, indicating a likely function in silk gland development (Fig. 3). The remaining 5 genes had relatively low expression in the silk gland. Therefore, only *BMGN011620* and *BMGN011783* were selected as the candidates for further analyses.

### *BMGN011620* has an expression pattern associated with silk protein synthesis

**Figure 4:**
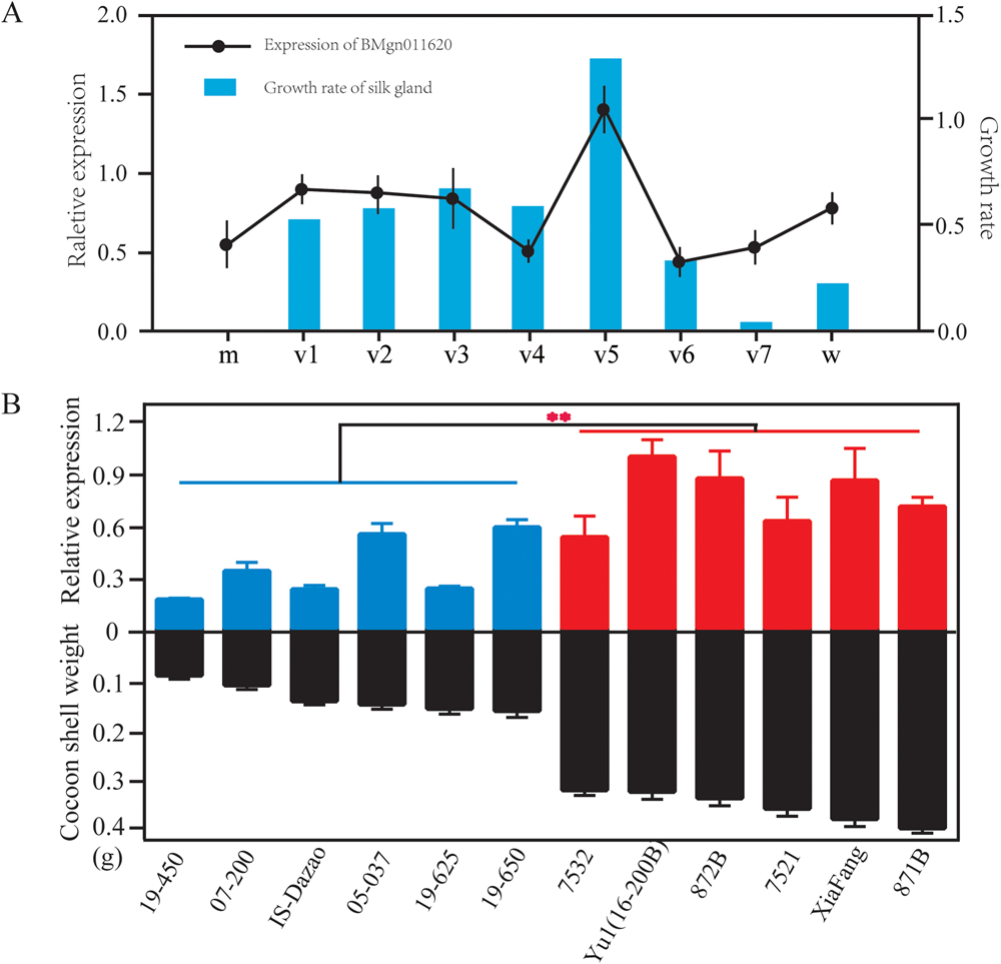
Analysis of the expression pattern of BMGN011620. (**A**) Association between the temporal expression of BMGN011620 and silk gland development in 5th instar larvae. Black line represents the gene expression pattern and the blue column indicates the growth ratio of silk gland on each day. ‘m’ represents the start of 5th instar; ‘v’ represents the day of 5^th^ instar; and ‘w’ indicates the wandering stage. (**B**) Association between the expression level of BMGN011620 and CSW in different silkworm strains. Blue and red columns (above zero) represent the expression levels of BMGN011620 in different silkworm strains, while the black columns (below zero) represent the respective CSW.

We first investigated the expression of *BMGN011620* and *BMGN011783* in the silk glands of 5^th^ instar larvae to determine whether their expression correlated with the development of silk gland. The results showed that although the gene expression levels were high in the 5^th^ instar larval stage, their expression patterns were different. The expression level of *BMGN011783* gradually decreased along with the development of silk gland (Supplementary Figure 6A), while *BMGN011620* had two expression peaks; one at the early stage and the second on the 5^th^ day of the 5^th^ instar (Fig. 4A). We then dissected the silk gland and determined its volume expansion rate on each day in 5^th^ instar. The results showed that the expression pattern of *BMGN011783* (Supplementary Figure 6A) was negatively associated with the absolute volume per day, while *BMGN011620* expression had a strong association with the increment of silk gland each day (Fig. 4A). Then, we detected the expression levels of the two genes in the silk glands of 12 silkworm strains with different CSW to determine whether their expression correlated with silk synthesis. The results revealed that *BMGN011620* was expressed significantly higher in strains with high CSW in comparison with low CSW strains (Fig. 4B). However, the expression of *BMGN011783* had no significant association with silk production in the tested strains (Supplementary Figure 6B). Thus, these results suggested that *BMGN011620* may be associated with silk protein synthesis. Since it encodes the 60S ribosomal protein, L18, in silkworm we named it *BmRPL18*. It locates in the region between 9,399,516 bp and 9,401,192 bp of chromosome 11 and only 23 kilobase (kb) downstream of marker647635 (Supplementary Figure 7A). cDNA cloning and sequencing showed no difference in the ORF of *BmRPL18*. Then genomic sequencing of *BmRPL18* detected 7 SNPs and one Indel at the upstream of the candidate (Supplementary Figure 7B) which may be one of the reasons why it showed varied expression among silkworm strains with different silk production.

### Differentially expressed genes show a clustering distribution in the genome

**Figure 5:**
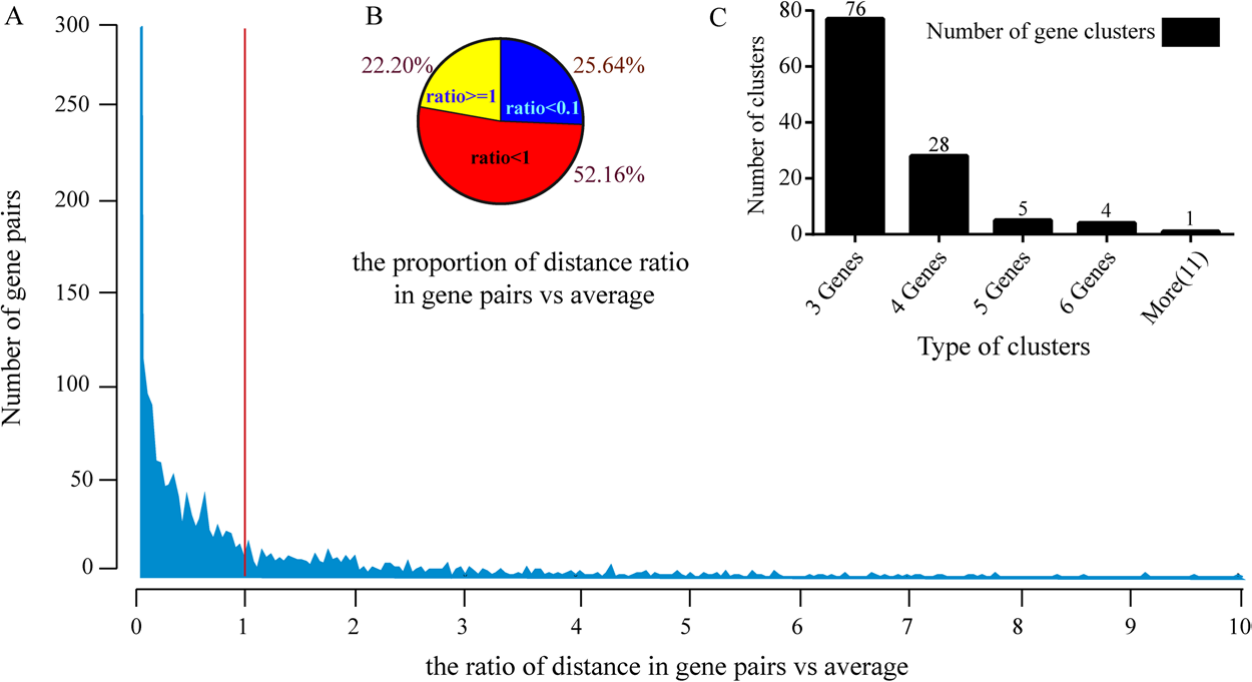
Clustering analysis of the differentially expressed genes (DEGs) in previous Omics studies. (**A**) Ratio of the practical interval between genes vs the expected distance. (**B**) Proportion of each distance ratio in gene pairs vs the expected. (**C**) Number of each gene cluster type.

Interestingly, out of the 8 linked regions, 6 were located on the 11^th^ chromosome and exhibit a clustering distribution on this chromosome. Genes with similar functions or with consistent expression pattern have been shown to cluster in adjoining genomic regions, which is an ubiquitous characteristic reported in many organisms^21–24^. Mapping results in this study suggested that genes regulating silk protein synthesis may also group into several regions of the silkworm genome. To confirm this, we collected the differentially expressed genes (Supplementary Table 8) in the silk glands of silkworm strains with varied silk production identified in previous Omic data^1,10,25^. We determined the distance between adjoined gene pairs to investigate whether they clustered together. The results showed that most gene pairs (77.8%) had a significantly smaller interval than the expected average (Fig. 5A and B), and 25.64% of the total gene pairs had the intergenic distance smaller than one tenth of the average (Fig. 5B). Then, we defined the gene cluster to three or more consecutive genes with intervals smaller than 30 kb (nearly average gene interval across silkworm genome). In this manner, 114 gene clusters (Supplementary Table 9) were detected and they distributed on each chromosome except the 18^th^ (Supplementary Figure 8). The gene number they occupied varied from 3 to 11 (Fig. 5C). We then select 4 gene clusters to validate whether genes in the identified clusters correlate with silk production. Expression level determination of them in silkworm strains with varied silk production showed that even though genes in one selected cluster have no association with silk production (Supplementary Figure 9); we did detect the other clusters containing more than one gene with significantly varied expression among silkworm strains with varied silk production (Supplementary Figure 9). Thus, these indicated that genes regulating silk protein production may tend to form clusters in the silkworm genome.

## Discussion

In this study, pooling sequencing-based linkage mapping was used to screen the genes associated with regulating silk protein synthesis in silkworm. This resulted in the identification of 8 loci, which is significantly higher than the existing reports based on traditional QTL mapping. The high density marker map is the likely reason for the greater mapping efficiency. We have previously analyzed the genetic basis for CSW by traditional QTL mapping and identified a QTL locus in the middle of the 11^th^ chromosome^7^. Composite interval mapping based mixed linear model (MCIM) predicted 3 significance peaks in this region. However, the low density of markers limited further analysis of this mapping region and grouped genome parts corresponding to these peaks into one single locus. In this study, we detected 3 QTL loci in this region that corresponded well with the previously predicted significance peaks. This result showed that enhancing the marker density within limits can significantly improve mapping precision. Besides, the development of a marker map with high density is also an advantage of the sequencing based mapping methods.

Notably, although more associated sites were detected, only one gene was identified to correlate with silk production through the association analysis of candidate gene expression patterns, silk gland development and CSW. Definition of the mapping regions may be the principal trigger to this result. To facilitate the gene screening efficiency, we defined the mapping region to 300 kb around the linked sites. Note that the individuals grouped in sequencing pools were from the BC_1_ population. Such grouping allowed only one recombination during gamete formation in F_1_ thus leading to a high linkage disequilibrium in the adjoining genome regions. Thus, the identified SNPs may not be exactly located in the region of the candidate gene but near the region with an artificial elevated significance caused by strong linkage, indicating that screening for genes around linked SNPs should be a requisite. The improved screening efficiency however, may result in missing several candidates in these regions. Furthermore, the criterion we used to identify candidates was to associate the gene expression pattern with the development of the silk gland. Omics data have shown a comprehensive physiological change during variety selection for silk promotion, including nutrients absorption, energy metabolism, protein synthesis and even immune reaction^2,7,8,10,25^. These involve many tissues besides the silk gland, such as the midgut and hemolymph, which are essential for the above physiological processes. Accordingly, genes involved in the development of these tissues may also regulate silk protein synthesis. Finally, other factors, including various non-coding RNA, are also indispensable for the development of organisms, such as the regulation of piRNA in sex determination of silkworm^26,27^. In our mapping regions, substantial numbers of non-coding RNA were predicted. However, whether these non-coding elements correlate with silk protein synthesis, although interesting, remains to be determined.

In this study, we identified *BmRPL18* that may regulate silk protein synthesis in silkworm. *BmRPL18* is a member of the L18E gene family and encodes the 60S ribosomal protein, L18, which is an important component of the eukaryotic 60S ribosomal subunit. A considerable number of ribosomal proteins are reported to regulate biological functions, such as development, apoptosis and aging through alterations of their expression levels^28^. RPL18 was shown to contain extra-ribosomal functions such as interaction with NS1 to regulate the translation and replication of Dengue virus^29^ and its requirement for the RNA translation of hepatitis C virus^30^. Besides, it has been reported that RPL18 can inhibit the double-stranded RNA (dsRNA)-activated protein kinase (PKR) to activate eIF2α and promote protein synthesis and cell growth^31^. These observations are indicative of the role of RPL18 in cell growth and proliferation. Note that the development of silk gland depends on the vigorous endomitosis of silk gland cells^32,33^. Studies have shown that the number, as well as size of silk gland cells varied in silkworm strains with different silk yield suggesting an association between the growth of silk gland cells and silk production^34,35^. In this study, BmRPL18 showed a difference in expression in the tested silkworm strains. However, it remains to be determined whether variations in the transcription levels of BmRPL18 can affect the silk gland cell growth. Moreover, the existing Omics data on silk production identified numerous ribosomal proteins to be associated with silk protein synthesis^1,8–10,25^. For instance, one study compared the protein components of different silk gland parts and found that the genes in the ribosomal pathway including BmRPL18 were highly expressed and enriched in this tissue^8^. Thus, both our results and the Omics data suggest that ribosomal components may play an indispensable role in the regulation of silk protein synthesis.

QTL mapping reported here, as well as the analysis of differentially expressed genes in Omics data indicated a clustering characteristic of genes regulating silk production. Genes clustering to a particular genomic region that regulated the same or similar physiological trait is a ubiquitous regulatory mechanism. For example, the synthesis of Alkaloid Noscapine, an antitumor from opium poppy, was controlled by a gene cluster containing 10 genes^22^. Another example is the supergene in the butterfly P locus, which controls the mimicry pattern^21^. Clustering distribution of genes is assumed to have been an efficient gene regulatory model during the evolution of organisms at least for some specific phenotypes. Studies have shown that the clustered genes always expressed a similar pattern although some belonged to different gene families^23^. This may involve the initial gene expression regulatory stage or change in chromatin structure. Highly organized chromatin will be first opened (open chromatin) before gene expression. These opened regions always comprise more than one gene thus leading to the simultaneous activation of their transcription^24,36^. If these genes were evolved to control the same physiological process, the regulatory efficiency will be greatly enhanced. The predominant determinant of silk protein synthesis is the development of silk gland. Therefore, it is rational to postulate that most genes that regulate silk production should be related to silk gland development, as well as have a similar expression pattern, suggesting a clustering distribution of this gene set. Our results have offered preliminary confirmation for this speculation. Meanwhile, whether genes regulating silk production cluster in genome and whether the gene clusters identified here correlate with silk protein synthesis remain to be verified experimentally.

In this study, by using pooling sequencing-based methodology, we deciphered the genetic basis underlying silk protein synthesis for the first time. Through a BSA-based linkage analysis and a series of association analyses between gene expression patterns, silk gland development and CSW, *BmRPL18* was found to regulate silk production. This is the first gene identified by forward genetic research related to this trait. Moreover, we have also shown for the first time that the genes regulating silk protein synthesis exhibit a clustering distribution in the silkworm genome. These results advance our understanding of the molecular mechanisms regulating silk production.

## Material and Methods

### Construction of pools for sequencing

To construct pools containing individuals with extreme phenotype we first developed the back-cross 1 (BC1) mapping population. Two silkworm strains, inbred Dazao (IS-Dazao) and breeder stock 872B, with contrasting silk production properties were selected as parents to produce the mapping analysis population; i.e., IS-Dazao × (IS-Dazao × 872B). Then, parents and the segregants in this population were reared at 25 °C and a 12 h photoperiod until the cocoon stage. Cocoons of each individual were sheared at the eye coloring stage to distinguish the sexes, and the CSW was measured in the two genders in both populations. Based on the phenotype data, male individuals with extreme CSW were selected in each moth area, respectively and the numbers of the individuals with extremely high and extremely low CSW (H-pool and L-pool, respectively) were about 10% of the total males in the mapping population. Then, the genomic DNA of parents and each selected individual were extracted and subjected to 1% agarose gel electrophoresis to check integrity and the concentration of each genome sample was measured by NaNoDROP 2000c. The genomic DNA of each individual was mixed in equal quantity to produce the pools for sequencing.

### SLAF-sequencing and polymorphism calls

The constructed pools, as well as the two parents, were subjected to standard SLAF-sequencing, which was performed as described previously^37^ with minor modifications. First, the reference genome of silkworm (http://sgp.dna.affrc.go.jp/pubdata/genomicsequences.html) was predicted using the software developed by Biomarker (Beijing) to select the suitable endonuclease pairs, *Rsa*I + *Hpy*166II. Then, DNA samples from the parents and the pools were incubated with the endonuclease pair at 37 °C for 3 h. After incubation, the products were subjected to polyadenylation at the 3′ ends, linkage of Dual-index sequencing joint, PCR amplification, product purification, sample mixture and glue recovery of products with the appropriate target length (294–364 bp). Qualified libraries were then subjected to sequencing on Illumina HiSeqTM 2500 at Biomarker Technologies Corporation in Beijing (http://www.biomarker.com.cn/english/). Then, Dual-index was used to analyze the raw data and to produce reads of each sample. After the joint sequence filter of reads, we evaluated the sequence quality and data size. Using the data filter method described by Sun *et al*., we obtained the high quality reads, which were finally mapped to the reference genome. By clustering the mapped reads, SLAF-tags in parents and pools were developed. Then, genotype of each reads in parents and pools were called to screen for polymorphic SLAF-tags and lastly to construct the polymorphic SLAF map covering the whole genome.

### Linkage analysis based on SNP-index

Genotype and the corresponding depth of each polymorphic SLAF-tag were investigated in the two pools. Based on the coverage depth of each genotype, the SNP-index of each SLAF-tag was calculated based on the functions below:

1 $$\mathrm{SNP}\_\mathrm{index}({\rm{aa}})=\mathrm{Maa}/({\rm{Paa}}+{\rm{Maa}});$$

2 $$\mathrm{SNP}\_\mathrm{index}({\rm{ab}})=\mathrm{Mab}/({\rm{Pab}}+{\rm{Mab}});$$

3 $${\rm{\Delta }}(\mathrm{SNP}\_\mathrm{index})=\mathrm{SNP}\_\mathrm{index}({\rm{aa}})-\mathrm{SNP}\_\mathrm{index}({\rm{ab}});$$

where “Maa” and “Paa” represent the depth of the homozygous recessive genotypes in the L-pool and H-pool, respectively; “Mab” and “Pab” represent the depth of the heterozygous genotypes in the L-pool and H-pool, respectively; by using Z-test, Δ(SNP-index) of each SLAF-tag was transformed to the corresponding Z-score. The function of Z-test used was:

4 $${\rm{Z}}=({{\rm{X}}}^{-}-\,{\rm{\mu }}0)/(S/\surd {\rm{n}})$$

“X” indicates the Δ(SNP_index) of each SLAF-tag, “μ_0_” the average of all Δ(SNP_index), “S” indicates the variance of Δ(SNP_index) and “n” the number of tested markers. Then, based on p = 1-NORMSDIST(Z_score) in EXCEL2010, linkage significance (p value) of each SLAF-tag was calculated and the corresponding FDR was corrected using Bonferroni. The SLAF-tags with FDR lower than 0.05 were defined to the linked markers.

### Confirmation of the putative linked markers

Indels in the 30 kb region centered on the linked SLAF-tags were screened. Based on their flanking sequences, specific PCR primer pairs were designed. By screening the parents, the positive polymorphic Indels were selected for the following linkage confirmation. Primer information for the selected Indels is listed in Supplementary Tables 4 and 5. Polymorphic Indels were genotyped in the selected individuals for pool construction one by one. In light of the genotype of each Indel, the Indel_index and the corresponding linkage significance were also calculated according to the functions in SNP_index. Then, the significance level of each Indel was compared with its corresponding level of SLAF-tag. If the p values were lower than the threshold above, the SLAF-tag was confirmed to the positive associated markers.

### Mapping region definition and gene screening based on EST data

Mapping regions for each associated site were defined to the 300 kb genomic region and centered on associated markers. Information of predicted genes, functional annotation and EST counts in these regions were investigated in the silkworm genomic database, including SilkDB and KaiKobase. Heatmap for the gene expression patterns was drawn by the heatmap program in R package. Genes with EST counts in silk gland and embryo were selected as candidates for further screening.

### Association analysis based on expression pattern

To confirm the expression pattern of candidate genes screened by EST count, various silkworm tissues were dissected out from larvae at the 3^rd^ day of the 5^th^ instar into cold normal saline (NS). The dissected tissues included wing disc, malpighian tubule, ovary, nerve, trachea, muscle, testis, midgut, anterior-middle silk gland, posterior silk gland, epidermis, fat body and hemocyte. The tissues were powdered in liquid nitrogen, and total RNA was extracted and purified using TRIzol (Invitrogen) according to the manufacturer’s protocol. Then, the first strand cDNA was generated by reverse transcription using the PrimeScript™ RT reagent Kit with gDNA Eraser according to the supplier’s instructions, and was used for quantitative and qualitative RT-PCR analyses. Then, silk glands were dissected from the beginning of the 5^th^ instar larval stage to the first day of wandering stage. For the association analysis based on gene expression pattern and CSW, silk glands were dissected from 12 silkworm strains with CSW variation at the start of wandering stage to ensure the same developmental phase. They included 19–450, 07–200, 19–200, 05–037, 19–625, 19–650, 7532, Yu1 (16-200B), 872B, 7521, XiaFang and 871B. CSW of these strains were measured as mentioned above. Total RNA and cDNA of each silk gland sample were prepared according to the above procedure and were then used for quantitative and qualitative RT-PCR analyses. The primers used for expression pattern confirmation of candidate genes were listed in Supplementary Table 6.

### Gene cluster analysis

Differentially expressed genes (DEGs) were gathered from the previously published Omics articles^1,10,25^. Overlapping genes in these reports were deleted to hold only one copy. The information of genomic positions for each DEG was collected from KaiKobase. According to the genomic position, intervals of each adjoining gene pair were calculated. Then, the total size of silkworm genome was used to divide the number of DEGs to get the expected interval of adjoining DEG pairs. Ratio of the practical distance to the expected was used as the rule to distinguish whether DEGs were distributed in a clustered manner. If three or more continuous DEGs had smaller than 10% of the expected interval then they were defined into one gene cluster. Silk gland dissected from 3 silkworm strains with low silk production and 3 with high silk production in the above association analysis between expression pattern and CSW were selected for the validation of gene cluster by q-PCR. The primers of these genes used for q-PCR are list in Supplementary Table 7.

### Data Availability

The raw reads obtained in this study have been deposited at NCBI Short Read Archive (SRA, http://www.ncbi.nlm.nih.gov/sra/) under the bioproject number PRJNA420782 and accession numbers SRR6347632, SRR6347633, SRR6347634, SRR6347635.

## Electronic supplementary material

Supplementary Materials

Supplementary Dataset 1

Supplementary Dataset 2
