# Supplementary material for: QTL analysis of cocoon shell weight identifies BmRPL18 associated with silk protein synthesis in silkworm by pooling sequencing
Source: Sci Rep. 2017 Dec 21;7:17985. doi: 10.1038/s41598-017-18277-y (PMC5740181; doi:10.1038/s41598-017-18277-y)
Supplement: Supplementary file 1 — Supplementary Materials [file 41598_2017_18277_MOESM1_ESM.pdf]

QTL analysis of cocoon shell weight identifies BmRPL18 associated with silk protein synthesis in silkworm by pooling sequencing

Chunlin Li[1,2], Xiaoling Tong[1,2], Weidong Zuo[1,2], Yue Luan[1,2], Rui Gao[1,2], Minjin Han[1,2], Gao Xiong[1,2], Tingting Gai[1,2], Hai Hu[1,2], Fangyin Dai[1,2]*, Cheng Lu[1,2]*

[1]State Key Laboratory of Silkworm Genome Biology, Southwest University, Chongqing 400716, China

[2]Key Laboratory for Sericulture Functional Genomics and Biotechnology of Agricultural Ministry, Southwest University, Chongqing 400716, China

*Author to whom correspondence should be addressed.

Supplementary Information

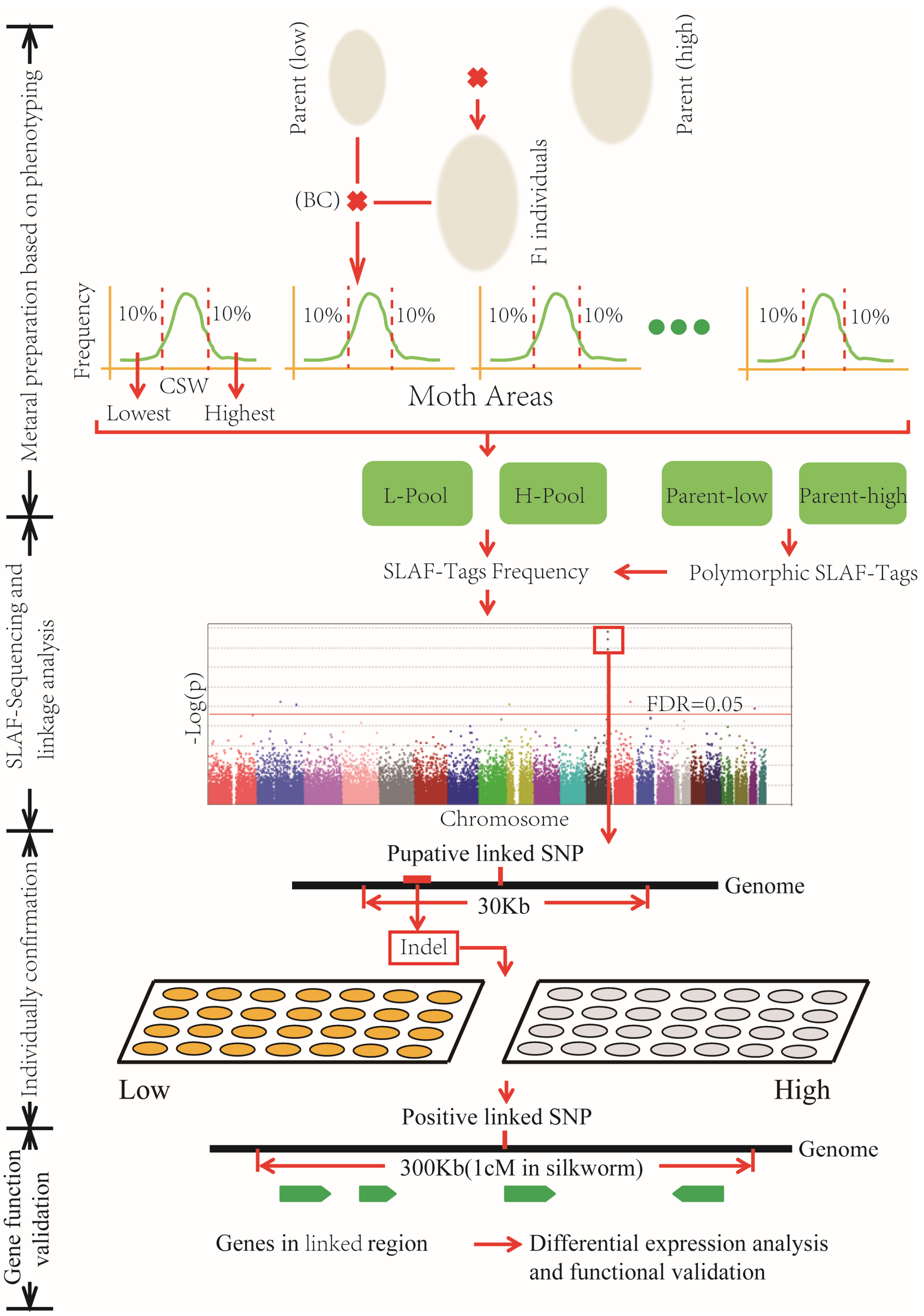

Supplementary Figure1. Simplified scheme of the pooling sequencing Based Methodology. The procedure could be divided into four parts. In the part for material

preparation, two silkworm strains with a huge silk production variation were selected to produce a series of moth areas compromised of back-cross offspring. The CSW of each individuals in these areas were investigated in order to select the individual with extremely low or extremely high silk and to form the pools for SLAF-sequencing; Linkage analysis part includes the SLAF-sequencing, polymorphic SLAF-tag calling and linkage analysis to lastly screen out the putative linked SNPs; then, the putative linked SNPs subjected to the following confirmation. In this part, Indels in the flanking 30kb region were firstly screened and then were genotyped in the sequencing population individually to get the positive linked SNPs. And in the gene function validation part, the linked region was defined and genes in the linked region were screened based on the association between expression pattern and silk production to lastly identify the candidate genes for the regulation of silk production.

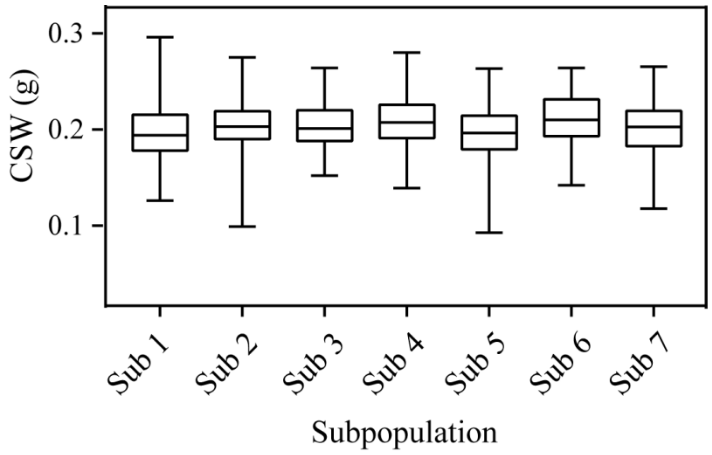

Supplementary Figure2. Measurement of the CSW of individuals in each moth area. Sub is short for moth area.

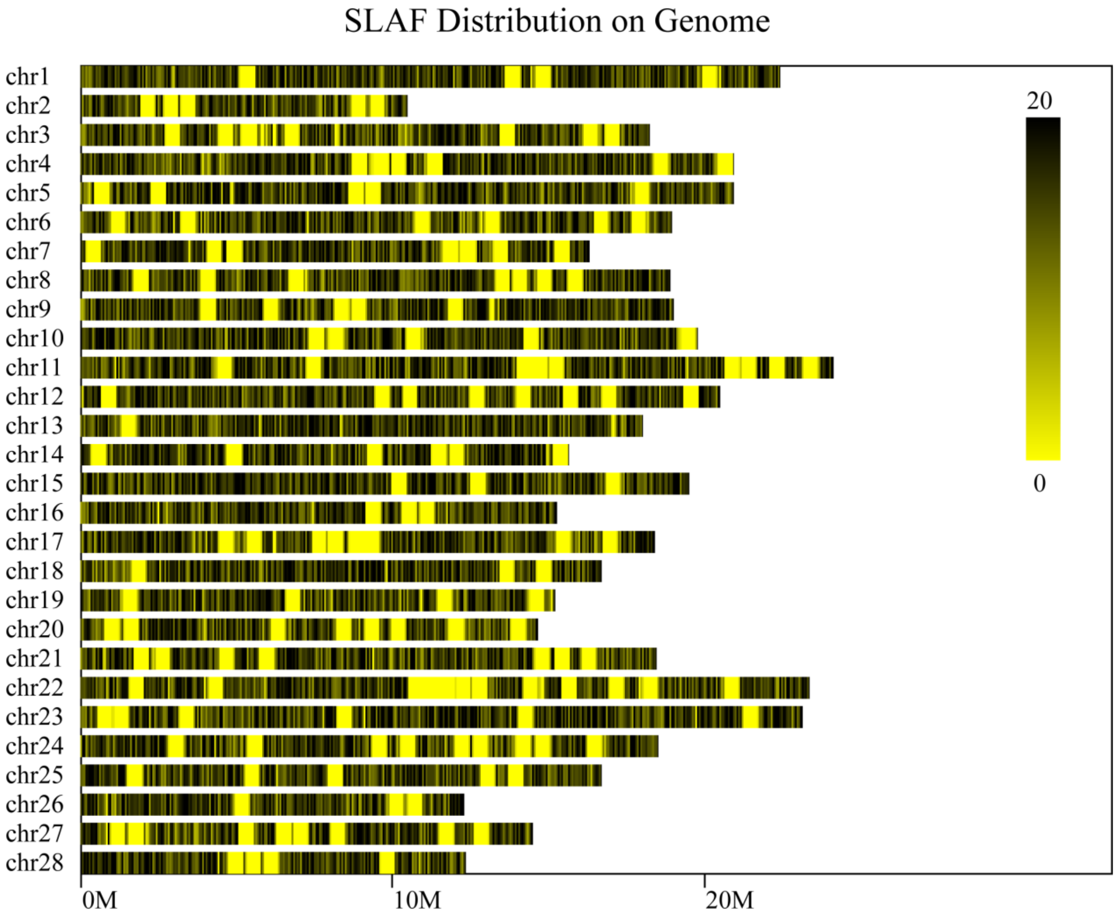

Supplementary Figure3. SLAF-tags distribution on each chromosome. The lateral bars represent the chromosomes of silkworm and black vertical lines on them means the SLAF-tags.

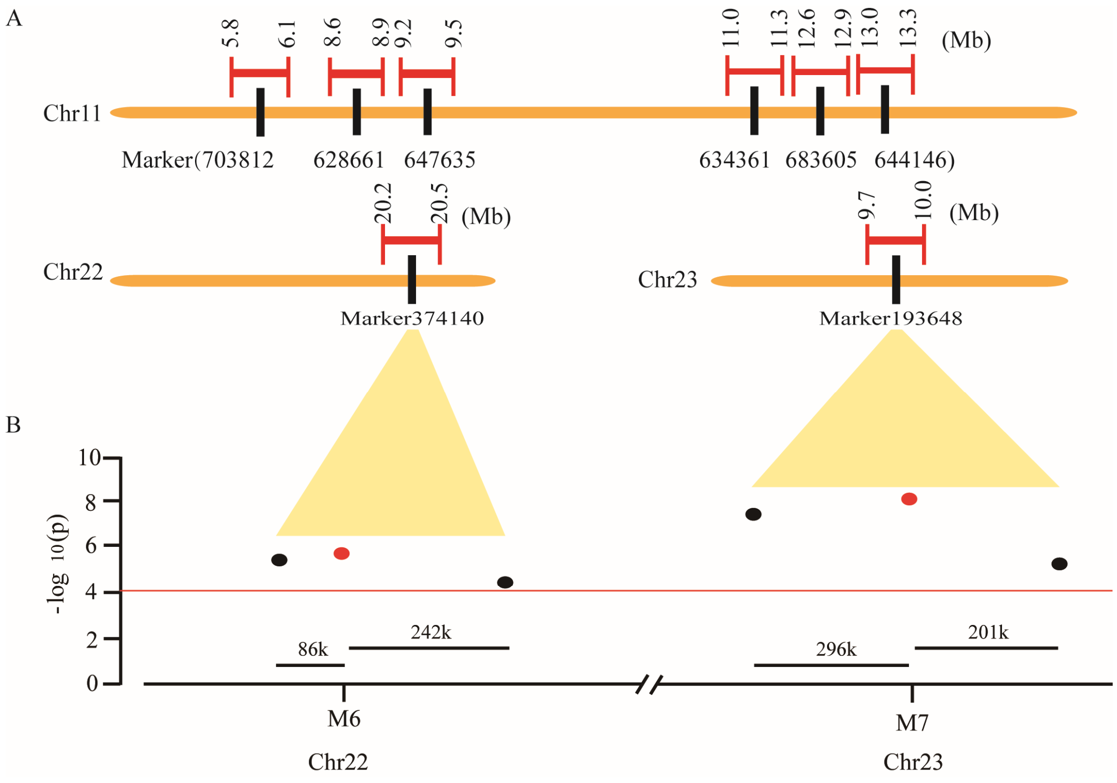

Supplementary Figure4. Distribution of mapping regions. A. the definition of linked region based on positive linked SNPs. The lateral orange lines represent the chromosomes containing linked SNPs, the regions limited by red lines represent the defined linked regions. B. confirmation of the QTL loci on the 22nd and the 23rd chromosome; the red points mean the linked SNPs and the black points mean the flanking Indels used for the linkage confirmation.

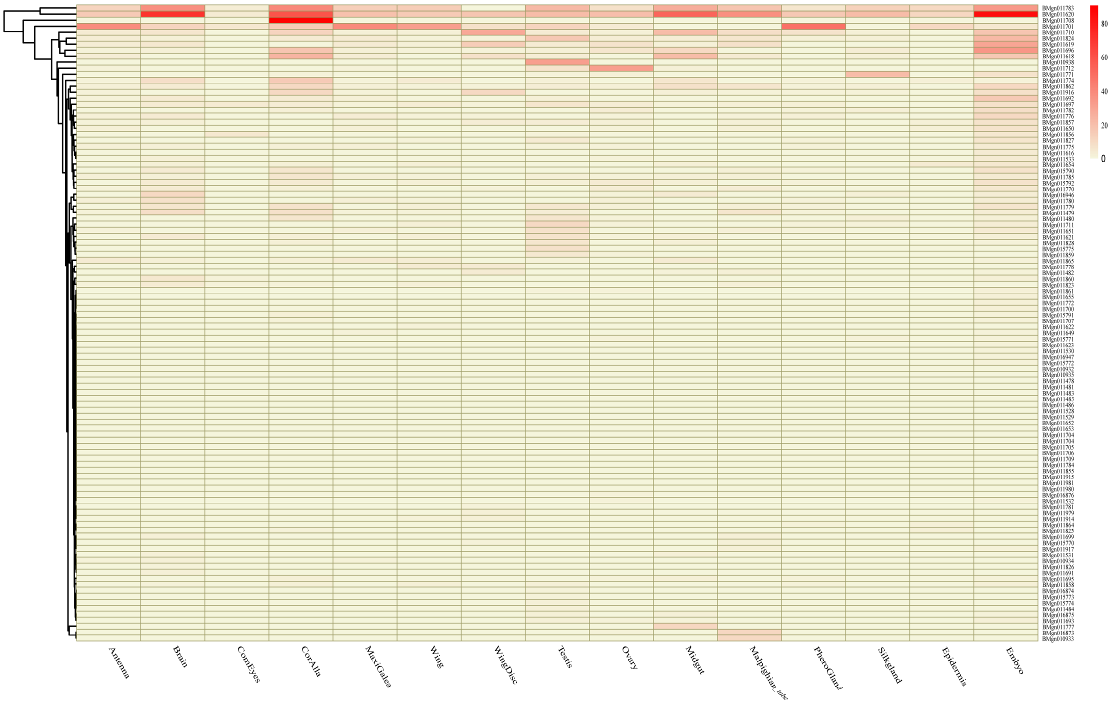

Supplementary Figure5. Expression pattern analysis of genes in mapping regions based on EST count.

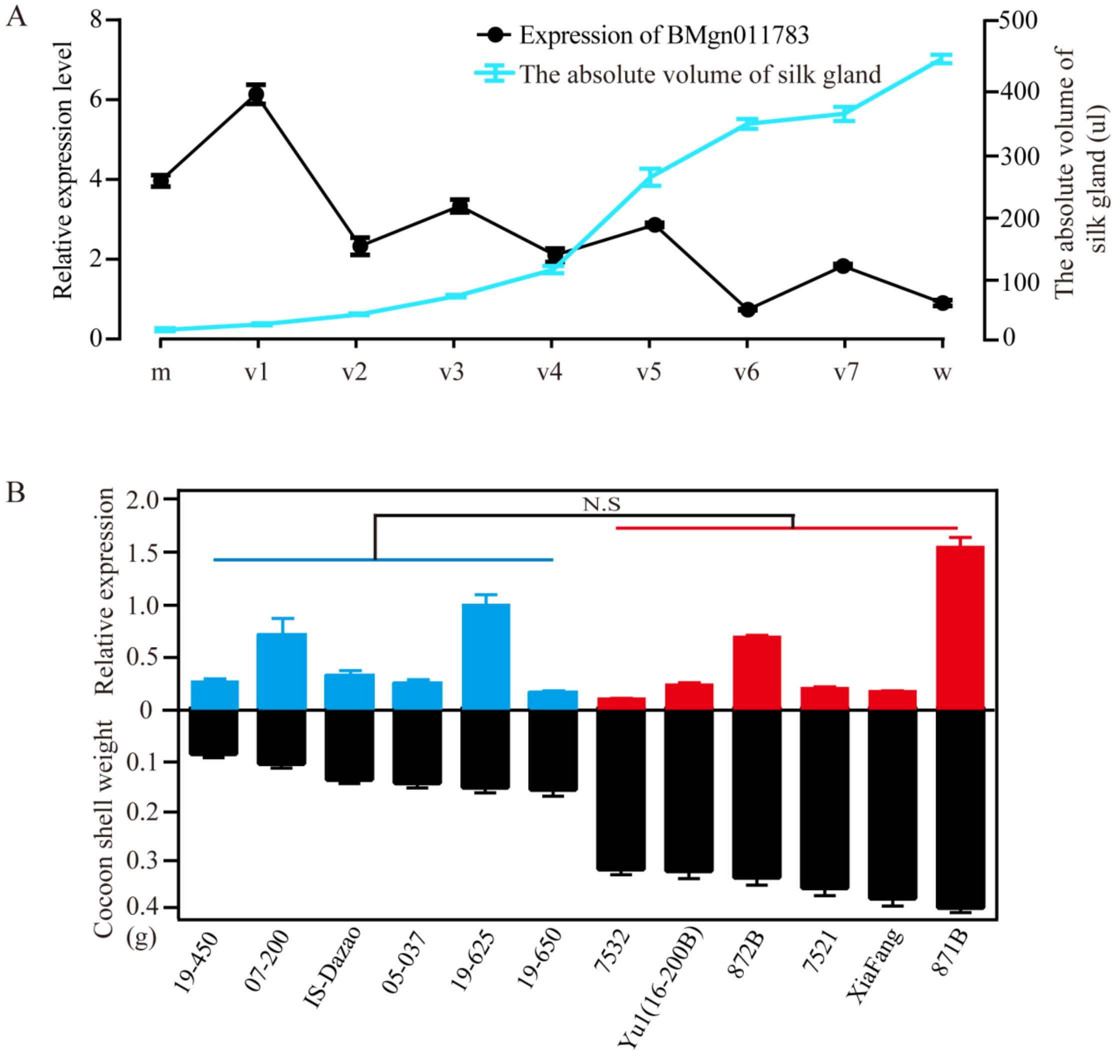

Supplementary Figure6. Expression pattern of BMgn011783. A. association between the expression of BMgn011783 and the development of silk gland; m represents the start of the 5th instar; the v1 to v7 means the 1st day to the 7th day of instar; and w means the wandering stage. B. association between the expression pattern and cocoon shell weight in silkworm strains with varied silk production.

A

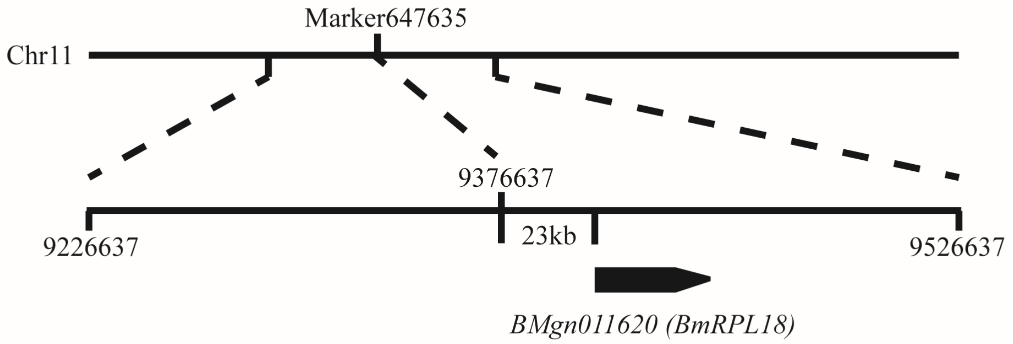

B

```
ACAAATTTGAGTGAACTTTTACGCTATCAAGAACGTTAAAAGCTCGCACTAAGCACACAGACACTAACGATT
AAAAATCTAGAACAATTTCTTCTAAAGGGCTGTTAATTTTATTTATTTTATTCTTATTTTATACTTATTGTACACA
                                                    T
AAAGAAAATACAATAAAAACAGATTTAAAGAATTCTAAATACTATGTACAAAGGCGAGCTTAACTCATTCATG
AGTTTTCTTCCAGCAAACCATTATCAGAGAGAAATACGATGTATAAGAGGGGAATAGTGTACAATATAAGAG
CTATTAAAGCATGATTTTTAACTAAGCATTAAAAAAATAATCTAACATTAAATGAAAAATACATATAAATAGAAA
TAATGTTAAGTTCAAATGTATTAATTAGTTACTTAAGCTATATCACTACAATAAAATAGGTAGGCGCATGGCGAT
TGTGAGGCTACTGGAATAACGGTCGAACTACTACTATTCAAATTAAAAGAAGTCAATAAAATTTATTTCCACTA
TTATAATCAACAATGCTAGATTAAAAGCTAATTTTGTGGGTTTTTTTGTATTAAATATTTATAATAAGTATGGTAG
GTATATAATTACTAAGACTAGCTATCCCAGGATCCACCAAGAACTAGTAATATGACAACACATCTGAAATTAATT
ATCCGCTAGTCTATGCCACGAAATAATTTTTATATTTAGTAAAATATGCACCTTGCCACCTAGTGATGCCGCGCT
AGCGTCTGAGCGCTGTTTATTCATTGGAGTGGTCATCTCCCATTGATTTCTGTCAGGATAGTACCGTTCTACTG
AAGCCATACGAGTACGTCCATTATATCCTCCAAGTGCGTATATCATACCGTCTAATATAAATATTTAATAATTTAA
GATTTAAATAGAAAACAACTTCGAATATAAAAAAGTAGGTATCCAAAGACTGATAACTACTACCTTAACCTAA
                                           A
GGAAAAATATTAATTGCCTATGGTTTTATCAGTTGAAAACAGTGCGGCTTAAAAGTCTAGTTACCAAGGTAT
A                                           T                    G
GAAATCTTTAAAGAATAAATCGAATCTCACGAGCGACCTCTCTAAATTAGACTACATTTTAATTAAGCAAAACC
AATATTAGGGAAAATAATAAAGACGATTATCGATTATTTTTTGGATCGAGAATCACATATTCTAGCAGGGAACT
GTTGCAATATACCGAATACCGCTGAGAATCAATGTACCTAAATTAAAATACTACACGAATGTTCATAGTTAAGT
ATCGTCAAATTTACAAAAATTGATAAAATTTCATAAAACTATCAATATCCCTATGATTTTAATCTAATACTGACCG
  T
TGCACGACAACGCTGACGTAGCATCTGGCTTGATACATACAGGCGCGTTCGTGCCACGTATTAGTGACAGGG
TCGTAACAACGTACCGTATTAAAATGGTCACTACCATCAAAACCTCCGATCATATAAATCAGATTGTTCAGTGT
GCAGAGGCCGTGATAAGCACGTGGAGTAAGGTCCATGTGAATTGAAAGAAACCAGCGGTCAGCTCTGAAA
TATATGAAGCTTTTATCATGCCTCCAAAAGCAGAACATCAAGCGAAATAATTTTCAGTTGCAGGTTAAATCATC
TCCTACATCAATTATACGATTATTTAATTTATTTTATTTATGCTTTGGCGAGGCTCTATTAATAAAAGCTCAGAAT
AGCTCTCTGAGTCTACATTTTGTTCCACACTACGCAGGCTTTCGAACTTGACGAAACTCTTAGAGCAATGTTA
AAACCGAACATGCACGGGCAGAAAATATGCGCCCGCATCCAAGTAGCAAATACAGAAAAGATTCAAGTACA
AATATTCACAGGATCTATGACGGCAACATGCTTGACCTACAAAACAACATACTCGCGTTTGTGCGAATTTTTAT
GTATGTCTTTATTCGACCAGTACCTAATAAGTTTTAATAGTTGAAACGACAAAGGTTTGAATGGCTTTCAATAA
AAATAGTTGGGTTTACTCTTTAAAACAGCTAATGAACGATATCATTGTCGATTAGTGCTTATAGTTGTTTGCTG
                                                        T
```

GCAAATAACTCAATAATTATATTATATATCTAGAGTAAGTATTGCAAGCATAAATTAAATACTGAATCAACCTTGT
ATCGTATGTTTCAACAAAGCTCGTAGGACTACCAGCACTCCATCCTCCAACAGCAAAAAGTATTTCAAAAGG
AATTCTAGGTCGCGCCAGTGGATCGTTTAAATCTGCTTCCATACCAGGTCTTGAATCTAGTAATGTTAAAAAC
ACCACTGCTGGGTATAACGCTTCTTGGCATTTCTGTAATAATTTTTTAAATTAACTATTATACATTTATTAGTCAT
CTCTAAGCTCAAAATAGAGAAATATATATTTACCACATCATCAACCACTGGCTGCCATTGTAATATTTTTGATTT
GAAATATTTAAAACTTATGTGACCAAATCTAACGCATGAAAGCAATGACGGAATGTACTTTCTCCTGTTTTCTA
AGTCATGTTCGACCCAAGTCTTTAAAGCTTGAAAAACAATTTCTTCATTTTTAACGTTCAGTTCGTCATCTCGT
AAAATGTCTTCCAATTCTTCGAAAGTAAGCGATTTAAATTCATTACATTCCTTTAGTATCTTATTAAAGTGCTGT
CTTATAAACATTTTCCCCTTCTTTTCTAATTCACCGCAAAAATAATATCGAGCAAATTTAAATATTCCTAGGCAA
TTATGAGGTTTTAATTCTTGCAGTAAGTACTGGCAACAAAGTTGGATAACTCCCACAACATCGAATTTGTCTG
CGTATGGCAGAAGATACTCAACATTCTCTGCTGTAACCTTACAAGTTCCAGTATAAGCGTAATCCAGTATTAGA
TTCATATAGAAACTAGGAACATCTACAAATATTTTAGTTTCCTCAGGTTCACCTTTGTTTAAAGAGTTTATAAAT
ATAGCTTTAAAATATGGGCTAACTGCTGATAGTATGGCGCGATGAACACCTATTGCTTTCAAATCCCTACACAT
TATCGTTCCATCACAGAGCTGACCGTTTTGTCGTAGTTCATTCCAAATTGCTGGGAATTCTACCACAGAATAAT
TTTCAGGAAGACACGCACATTTTCTTTGTCTCGTGATAATTTTCTTTTTGTTTAAACGTGATCTCGCAATTGAA
C

GGCGTACGTACAGAATTTTTGGCTTTTGTTAATTTTTTATGTTTCGTATTAAATTCCATCCCTTTTGAAAAAAC
GGCGGTCGAAACTAAATTGTCCTGTTGACATAACTAAAGTCTTTCCTCACTTGTTTCATAACCGCGAGAGTGA
CATTAAAAAGATCTTTATGAGGAGAATGACATATTGGATTCTGTTTTAAACTTAAAAGATCCCCGCATCTTTAA
ATATATACATATATGTAAATAATAATAAGAAATTATGAGAAAACAATCTTGATGATAACTTTAACGTAAATAAATC
TTTTTTATTTTTTGGTCAGGAGGAAACCGCCTGACTTCCACCTACGTGTTAGGAATGGTAAGCGGAGCTTGT
CGCAGTTGAACTGACTGCTCTCACTGAAACCTCCCCGTTGCTCAAAAACTTGCGTCGGAGCCCTGAAACTCA
TTTATTCAATGCTCTTGACACTTTGAGTTCGAAGTCTGCCCTTTTTATCCTTACCTAATCACTAGTAGCCTGAG
ACTCTATTCCAACTACGTGTGAACGGTAGGTGAACTCACGAGCTTAACCTGTTTGGATTTATTAACACTAACC
CCAATACGAGGAGTCTTTCGCAGAATCTACGACAGTATCGGAATCGCGACCCATTGAGAAGAACTGGTGAA
AAACTCAGTGAGTATATCTATTGGTTAGATCGAAGTCGTCGTCGTCATCTATAAGATCGACGAGGACAGTCAC
CAGTACGTGAGGAAAAATCGCAGAATAATTGCAATGATTAAATTTAAGTTCAATAAAACCTCCAAAATTACAA
ATTTAAACATGAAGAAACACTATTAAATAAACAAAATAAAATAATTCAACAACTTCGCTCCTGCCAGAGTGTA
AGATTGCCACACCAAATATTTTATTGGTAACACCAACATAAACTGTCAAACATTTTGATGTATCTAGGATACTCT
GTTCTATTGTCCATAGTCTCTTTTTCTTCTGGTTGTCAAGATGGTGAGTTTTGCCAAACGAATAAATAAATAAA
TCTTTAAAAATCCAAGTGAAAATACAGTGATGTGAAAGTTCAAAGTGATTTTAATTACGAAACTGTGATAATT
ATGTGTTCTTATGAATATTTCAGGGTATTGACATCAATCATAAACACGACAGGAAAGTTCGGCGCACCGAAGT
TAAATCTCAAGATATTTACTTGAGGTTACTTGTAAAGGTACTTACGATTAAATGTTTCATTATAACTAGCGATGT
TACTTAAAATTTTATTTGTTATTATACGCACATAGTTTGTCTGCGAAAATTGTTGTAGACCTACGATAACCTCAA
CTATTTGTCCACAGCTTTACAGATACTTGGCCAGACGTACAAATGCCAAATTCAATCAGATCGTTCTACGCCG
GCTCTTTATGAGCCGTATCAACCGGCCACCAATCTCTGTGTCTCGTTTGGCGCGCCACATGAAGAAGCCAAC
TCGTGAGGGTTTGATTGCCGTGGTAGTGGGGACAGTCACAAATGACGTGAGACTGTACAAGATACCGAAGA
TGACGGTGGCTGCTCTTCATGTTACCGAAAAAGCTCGTGCACGCATTTTGGCTGCTGGAGGTGAGATGTTTG
TTTTTTCTGCCAATAAGGTTATAAATCATAGAAATAACAGAAATAAAATCGTTCAGGAATCTCCGAAAAAATA
AATTAACGTCCAGAATTTACTATATAAGGCAATACAAGTTTATTAGATTCTGAGAAAAACAGTTGAACTTATCC
ATGTTATAAATCTCATGCTCAAAACTAACAATTTTAGCTCACTATTATTATATTGTGTTCAATGTATATTTAGAGA
CTCCCATTTACTATCTATTATTGTTAAGTCCATAAAATAACTCTTCTGATACTTTAAAAGGAGTAAATAATAACAA
GTCTGTTGGATGTGAAAAGATCATTTAATAATTTTATATAAGATAATTATATTGTAAATTGATAATTAACTTTGAT
AATTAATTTGAAAAAGAGATTTGTTATAGATCAACACTATAATTTTATTTTATGAGCACCAGTGAAGCCAATTG
TAAGAAAAAAAAATATTTTCTTAATGCTTCTACACTACTACTAACAAACTATTTCTAATTTAAATTATTACAGAGAT

ACTATTACAGAGTTTATTCTGATCAGTAAAAAAAGAAATTTGCCAGATTGGTTTATCAATTGTTGATGGTCCAT
CAAACATATGGCAATTTATATTTTTATGTAAATAAATATTAATTTTAATCTTTGCAACGTAAGTTAGGCCATGTCA
ATTTTTTTAAAACATCTTTCAAAGATGTTATGATGTTTCTGTTTGATTGTGTATCTGTAATGAAAATTGATTTCA
ATTTTCTGTATTTACAGGAGAAATTCTTACTTTTGATCAGCTGGCTCTTCGTGCTCCGACTGGCAAGAAGACA
GTACTGGTACAAGGTCAGCGAAATGCTCGTGAGGCAGTGCGTCACTTTGGCCCTGCTCCAGGAGCACCGCG
CTCTCACACTAAACCCTATGTTCGCACCAAGGGACATGAAAAAGCAAGGCCCAGTCGTCGTGCTAATGTCTA
ATTTTTAATATAAGGACTACACACCCTAATTACTTGGTATTATTTTATAATTGAATAGTCCCAACAACACTAACA
AATATTTCGCCTTTTATATGTACTAATTGATCTAGTGTTGTCTTTATAGTAGATAATAATTAATTACCTTTGATAGG
CAGACAATGTAAATGATATCAACTTGATGTGGGTGATTGCCAATTCTCTATATAATAAGCAATATTTATCAGCTA
AATTGTTGACACTACTATTGTTTAACTCATTGAACACTATGACGGATACCATGTGGCCACAGTAAAGGCCGCA
CCAATATATCAAGTGTCCATAAATCATAGACGTGAATATTCCGAATATTGACCAAAAATAACGTGACT

Supplementary Figure7. Information of *BmRPL18*. A. the genomic position of *BmRPL18*. B. the genomic sequence of *BmRPL18* in parents. The continuous nucleotides are the sequence of it in IS-Dazao and the nucleotides just below show their corresponding genotype in 872B. the regions with line mean the exons.

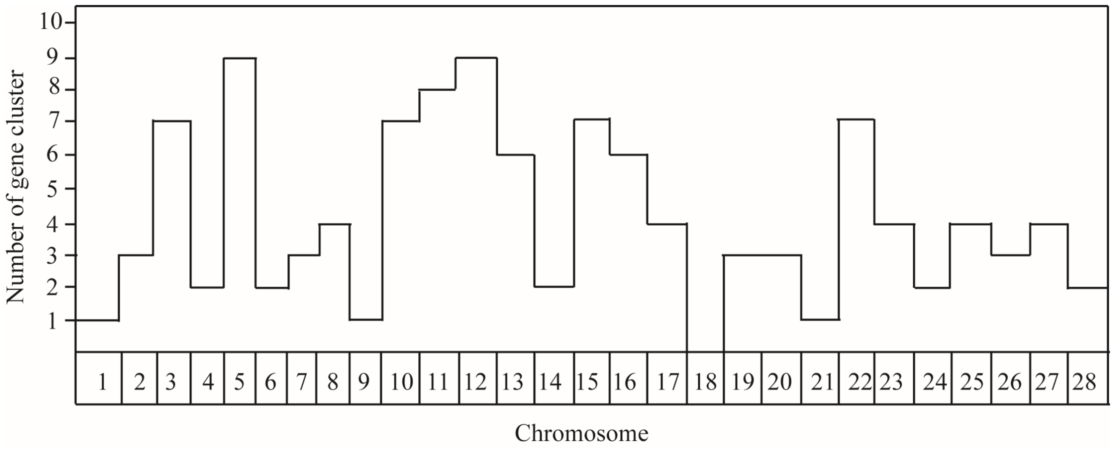

Supplementary Figure8. The number of gene clusters on each chromosome.

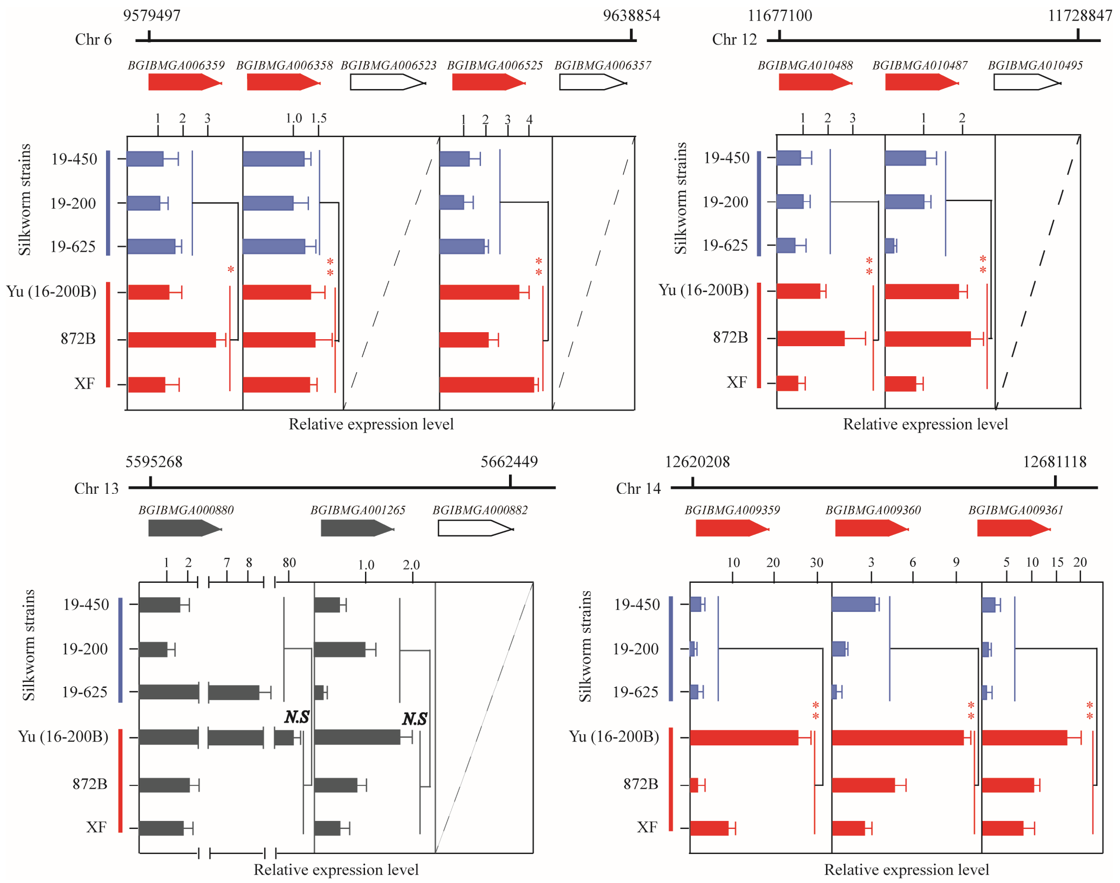

Supplementary Figure9. Validation of gene cluster by expression pattern determination in silkworm strains with varied silk production. Four gene clusters were investigated and showed here. For each of them, the upper line showed the genomic region of the cluster, genes below are the members of this cluster. And the bottom box showed the expression of the corresponding gene. Genes with red filling mean that they have an associated expression pattern with cocoon shell weight; the genes with grey filling means they have no association with silk production and the genes with no filling mean no expression detection in silk gland. The strains with blue line right are the silkworm strains with lower silk production while the strains with red line right are them with higher silk production.

Supplementary table1. Individuals with extreme CSW selected from each moth area for pooling

No. of moth area	Number of individuals	Lowest[1]	Highest[2]	Ratio of individuals (%)[3]
1	154	15	15	(9.7+9.7)
2	152	15	15	(9.9+9.9)
3	157	15	15	(9.6+9.6)
4	127	12	12	(9.4+9.4)
5	160	16	16	(10.0+10.0)
6	174	17	17	(9.8+9.8)
7	157	15	15	(9.6+9.6)
Total	1081	105	105	(9.7+9.7)

[1] number of selected individuals with lowest CSW in the corresponding moth area;

[2] number of selected individuals with highest CSW in the corresponding moth area;

[3] ratios of selected individuals to the total in the corresponding moth area;

Supplementary Table2 Distribution of developed SLAF tags and the polymorphic SLAF markers

Chromosome ID	SLAF number[1]	Polymorphic SLAF[2]	Hot SLAF[3]
**Chr1**	5,956	788	602
**Chr2**	2,285	234	180
**Chr3**	4,002	368	286
**Chr4**	5,126	592	428
**Chr5**	5,378	478	267
**Chr6**	4,480	494	422
**Chr7**	3,761	324	264
**Chr8**	4,460	465	333
**Chr9**	4,838	446	325
**Chr10**	5,038	600	428
**Chr11**	5,745	695	552
**Chr12**	4863	541	362
**Chr13**	5114	560	460
**Chr14**	3783	242	148
**Chr15**	5378	533	444
**Chr16**	3925	401	306
**Chr17**	4084	423	277
**Chr18**	4458	507	287
**Chr19**	3798	394	312
**Chr20**	2982	260	195
**Chr21**	4333	346	216
**Chr22**	4978	567	416
**Chr23**	6081	627	494
**Chr24**	4025	350	258
**Chr25**	4128	426	352
**Chr26**	3145	278	202
**Chr27**	2994	289	233
**Chr28**	3082	228	94
**Total**	122217	12476	9143

[1] the number of developed SLAF tags in corresponding chromosome;

[2] the number of polymorphic SLAF markers in corresponding chromosome;

[3] the number of selected SLAF markers with the coverage depth above 5× in corresponding chromosome;

Supplementary table3. Genes with EST counts detection in silkgland and embryo.

Gene ID[1]	Functional annotation	Silk gland[2]	Embryo[3]
BMGN016946	Succinic semialdehyde dehydrogenase	2	3
BMGN015771	Putative uncharacterized protein (Fragment)	1	1
BMGN011916	DUF846 (Eukaryotic protein of unknown function)	2	10
BMGN011862	Putative uncharacterized protein	1	8
BMGN011824	Acetyltransferase (Fragment)	2	22
BMGN011783	Autophagy related protein Atg8	13	34
BMGN011782	Putative uncharacterized protein (Fragment)	2	9
BMGN011771	TRM112-like protein (Function unknown)	21	7
BMGN011710	Ubiquinol-cytochrome c reductase core protein II	2	18
BMGN011696	Histone deacetylase 2C (putative)	3	35
BMGN011618	Cytochrome c1	2	17
BMGN011620	60S ribosomal protein L18	21	84

[1] the gene ID in Kaikobase;

[2] the EST counts detected in silk gland;

[3] the EST counts detected in embryo;

Supplementary table4. Information of Indel markers for linkage confirmation.

Name of primer	sense	anti-sence
marker1	GTTCACGAATCGCCCTACTA	GTGTTTGTTGCTGGCTACGA
marker2	GACCGAGCAGCACGACA	CTGCGAGGGAGTACGGTT
marker3	GACAATGACTAACATGCCAATG	CTATGGGTTATACACTGCTGCT
marker4	GAGTTTATGAGAAGAGCGACG	AGCGTGAAGCGTTAAATCAA
marker5	AGCCCCTAATCATTATGGTACA	ATGTATGTATATGCTTGCTTGATG
marker6	CTCACGGGAGGACTGAAGAT	TGCCTACCGTTGCCTATCTA
marker7	TCCCAGCGACCATTATACAA	GAAGTTGCTCACAATGGGC
marker8	GCAGTAGGCAGCCATGATAA	AGAAGCAAAGCGAGGGC
marker9	ATGCGATTTCATAGGATTTGT	CAAGTAAATACTCACATCCTGGC
marker10	GAAGAAACCGTGAAGGGATT	AGCAACAGATAGCCCTAAAGA
marker11	CATTTACGTGAGCGTGTCTCT	CTTCTTTAGAATCGTCGTCAAC
marker12	AAAGGGCTTCGTAGTCAGATG	CGGCTACCGTATCAAGACAC
marker13	AAAATGACGAGGATAGTTAGGTC	TTTCAAGCCAAATGTTCCAA
marker14	AAAGTATTCGCATTGCTCTGA	GGCGGTTTCGCTTATCAT

Supplementary table5. Information of Indel markers for linkage confirmation of QTL regions on the 22nd and 23rd chromosome.

Name of primer	sense	anti-sence
Marker6-1	TGCCTGCTCCTAAATCACTG	TGTGTTCTATGATGGCTACGG
Marker6-2	TCTCCTGGGGTGGTAATGTT	CGAAATCACTGCTGACGATAATA
Marker7-1	TTGGAAAGGTTCGCCAGTAT	AAGTTACCGAGTGGACGCC
Marker7-2	GGCAGGCATCACCATCC	GCCGCCACCCATCTTTA

Supplementary table6. Information of primers for real-time quantitative PCR.

Name of primer	sense	anti-sence
BMGN011618	CCGTCGGTAGGATTTGTGG	AGGGTTGTGAGGTGGGTGAG
BMGN011620	CGCCTCTTTATGAGCCGTATC	ACCAGGACTGTCTTCTTGCCA
BMGN011696	GTCGGTCTGTCTCGCTATCGTA	TCCTCTTCTCCTTCACCCTCG
BMGN011710	CGTTATGAACCACAAGCCGA	CTATTCCAGACGAGGAGCATTATC
BMGN011824	TGTGTCAGATACGCCTCCAGC	TCCTCACCGTCCTCTTCCATT
BMGN011862	TGGTGTGGCTGATAAGGTAACTG	TCTTCTTCTAATAGGAACTTGTGCG

Supplementary table7. Information of primers for gene cluster confirmation.

Name of primer	sense	anti-sence
BMGN006359	TGTTATGCCTGATGGTGGAGC	TTGTTCTTGGAAGCCTTTCTGTTA
BMGN006358	GCCTTGGAATACCGAATACAGC	GATTGACAACATTGCCTTGAGAA
BMGN006523	TTTGCCGAGTTCCAGGGTTA	TTCCGCTTCTGTCTCCGTG
BMGN006525	AAGCGGTGCGTTGTTCTGTA	GCATTGCCTTTGCTGTTCATA
BMGN006357	TCCCAAGATGAGCAAGACACTAAA	TGCGTTCCAACAAAGGAGGTA
BMGN010488	TTGAAACTCCTGCTTTACAACGA	TGGCTCACTCATTCTTCCGAC
BMGN010487	AAAGTCCAAGGAAAGGCACG	TCACACTCAAGACGGAGCACA
BMGN010495	TATCCTGGCATAATCGTGATCC	TGAACTGAACCCGTCTCCGT
BMGN000880	TGACCATACTGGACCTCGTGC	TCTCCTCGCCCATTGATTGT
BMGN001265	AACTTGCTTGTCGCTCAGAAAC	ACTAACGGTCCTATCCTAATGGCT
BMGN000882	CAAAGCACAGAGGGTGGAAAG	TCATAGATGGTCTGGTCATAGCG
BMGN009359	AGGTTCTTATGTCAGCAGGCAA	CGTCCGTCTCCAGAGGTATCA
BMGN009360	ATGCTCTTTGGGACGATTACG	AGTTATTCTTCGCCTGTTGGTGT
BMGN009361	ATTTGATTGTTTACCTGACTGGCTA	TCTCTGCTTCCTTGGCTGATG
